# A causal relationship between panic disorder and risk of alzheimer disease: a two-sample mendelian randomization analysis

**DOI:** 10.1186/s12888-024-05624-3

**Published:** 2024-03-04

**Authors:** Yueqin Tian, Qiuping Ye, Jia Qiao, Lian Wang, Yong Dai, Hongmei Wen, Zulin Dou

**Affiliations:** 1 Department of Rehabilitation Medicine, The Third Affiliated Hospital, Sun Yat-sen University, No. 600, Tianhe Road, 510630 Guangzhou, Guangdong China; 2 Clinical Medical College of Acupuncture, Guangzhou University of Chinese Medicine, 510006 Guangzhou, China

**Keywords:** Panic disorder, Alzheimer Disease, Mendelian randomization analysis, Causality, Genome-wide Association study

## Abstract

**Background:**

Observational studies have suggested a link between panic disorder (PD) and Alzheimer disease (AD). This study aimed to identify the underlying association of PD with the risk of AD using Mendelian randomization.

**Methods:**

Genetic instrumental variables (IVs) were retrieved in the genome-wide association study between PD and AD. Then, five different models, namely inverse variance weighting (IVW), weighted median, weighted mode, MR-Egger and MR-robust adjusted profile scores (MR-RAPS), were used for MR Analysis. Finally, the heterogeneity and pleiotropy of identified IVs were verified by multiple sensitivity tests.

**Results:**

The Cochran’s Q test based on MR Egger and IVW showed that no evidence of heterogeneity was found in the effects of instrumental variables, so a fixed-effect model was used. IVW analysis (OR 1.000479, 95% CI [1.000147056, 1.000811539], *p* = 0.005) indicated that PD was associated with an increased risk of AD, and a causal association existed between them. Meanwhile, weighted median (OR 1.000513373, 95% CI [1.000052145, 1.000974814], *p* = 0.029) and MR-RAPS (OR 1.000510118, 95% CI [1.000148046, 1.00087232], *p* = 0.006) also showed the similar findings. In addition, extensive sensitivity analyses confirmed the robustness and accuracy of these results.

**Conclusion:**

This investigation provides evidence of a potential causal relationship between PD and the increased risk of AD. Based on our MR results, when diagnosing and treating patients with PD, clinicians should pay more attention to their AD-related symptoms to choose therapeutic measures or minimize comorbidities. Furthermore, the development of drugs that improve both PD and AD may better treat patients with these comorbidities.

**Supplementary Information:**

The online version contains supplementary material available at 10.1186/s12888-024-05624-3.

## Introduction

Dementia is an acquired progressive cognitive impairment, which is the main cause of incapacity, disability and death. Currently, it is estimated that 50 million people in the world struggled with some forms of dementia, and with the aging of the population, 139 million people around the world are expected to suffer dementia in 2050, which will bring an even greater burden on the socio-economic and health systems [1–3]. Alzheimer disease (AD) is the most common type of dementia (60–80% of all dementia cases), which is characterized by neurofibrillary tangles and the formation of neuritis plaques [4]. Patients often show memory disorders and cognitive deficiencies in other areas [5]. The main risk factors include age, genetic susceptibility, anxiety, depression, environmental pollution, lack of physical activity, and low academic performance [3, 6–8]. 50-80% of people with Alzheimer disease have mood disorders, including insomnia, anxiety, panic disorder, pain, and hypochondria [9, 10]. Panic disorder (PD) is an anxiety disorder characterized by recurrent panic attacks [11]. In the general population, the lifetime prevalence rate of PD is between 2.7% and 4.7%, which may be related to smoking, asthma, etc. [12, 13].

It has been early recognized the notable similarities between PD and AD. Firstly, there is strong genetic susceptibility to both PD and AD [14]. Secondly, observational studies have found that AD patients are often comorbid with PD, phobias, and even anxiety, and that the quality of life of patients with these comorbidities is severely affected [15–17]. In contrast, patients with PD scored worse in all cognitive areas, and they performed worse in memory tasks [18]. That is, patients with PD were more likely to develop cognitive and memory disorders. Finally, it was also found that PD and AD may have similar pathophysiological processes. For instance, 5-HT1A receptors were closely related to the pathophysiology of both PD and AD. 5-HT1A receptor binding levels in the cerebral cortex and amygdala were lower in both PD and AD patients [19–21], and in the AD patients, its levels of the cerebral cortex were negatively associated with the severity of dementia [20]. Thus, the 5-HT1A receptor has been identified as a shared pharmacological target for both PD and AD. However, one study found no significant association between PD and any cognitive fields [22]. In short, this evidence was uncertain as to the relationship and specific direction of the link between PD and AD. In order to further understand the interaction between PD and AD and to propose clinical diagnostic and therapeutic strategies to improve the comorbid population with PD and AD, it is necessary to clarify the potential causal relationship between PD and AD.

Although a potential link between PD and AD has been found, observational studies are unable to provide direct evidence of causality because of the obvious limitations inherent in observational studies, such as reverse causality bias, measurement error, and confounding. Mendelian randomization (MR) analysis is an analytical method for causal inference, which is used in the field of epidemiological etiology [23]. By using genetic variants as instrumental variables and relying on uniformly, randomly, and independently distributed genetic variants during meiosis, MR effectively avoids confounding and reverse causality [24]. In this study, we used MR analysis to study the causal relationship between PD and AD by using germline genetic variation as an instrumental variable (IVs). In order to obtain fair results, three hypotheses of MR must be satisfied [25, 26]: (a) correlation hypothesis: genetic IV should be directly related to exposure; (b) independence hypothesis: genetic IV has nothing to do with confounding factors related to selected exposure and results; and (c) exclusion of limitation hypothesis: genetic IV affects results only through exposure rather than through other biological pathways. Here, we try to use a two-sample MR analysis to assess the causal link between PD and AD.

## Methods

### Study design

**Fig. 1:**
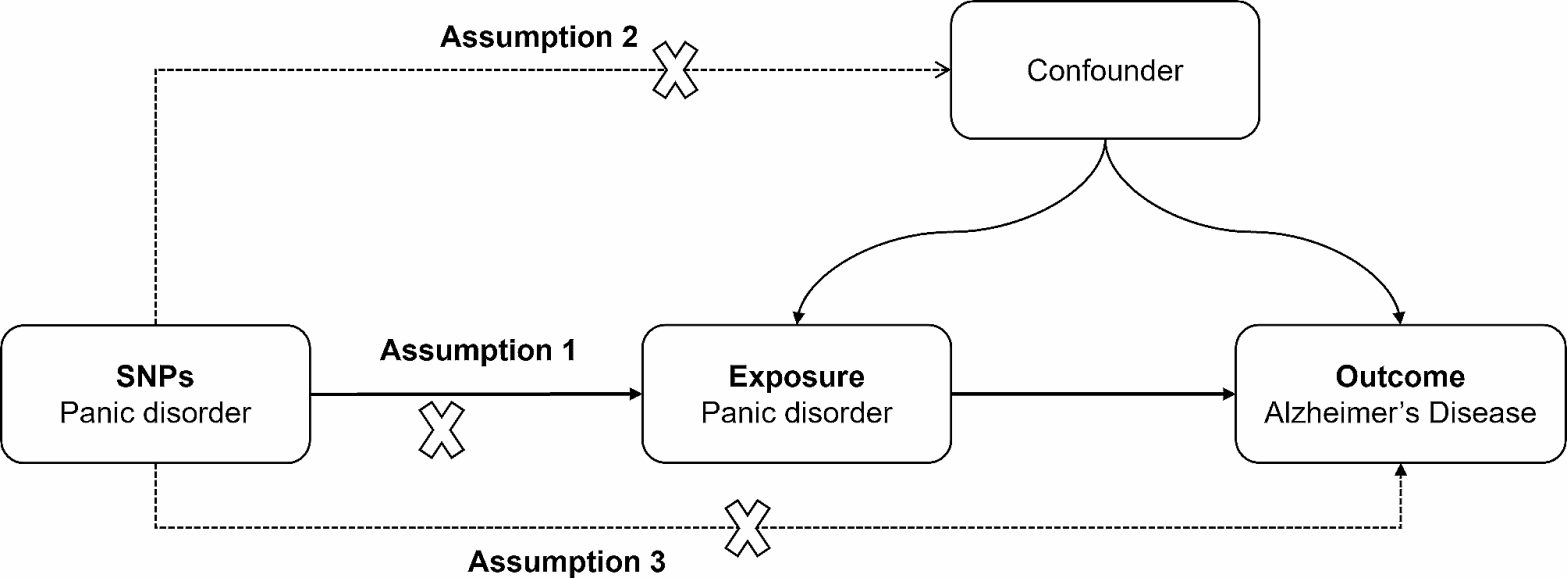
The principles of MR analysis

Two-sample MR is thought to be an approach to identify the causality among exposure and outcomes through the use of exposed genetic variants as an IV. The Principles of MR Analysis are shown in the Fig. 1. This approach allows access to available public data sets of “exposure” (as a risk factor) and “outcome” (as a disease) from large sample genome-wide association studies (GWAS) and bridges the deficiencies of observational research. This research is the second data review of the available databases. In the present work, a two-sample MR analysis was used for assessing the causality between PD (exposure) and the risk of AD (outcome).

### Data resource

To obtain a qualified exposure and outcome dataset, we retrieved publicly available GWAS database, and thus no additional moral approval was required for this study. Considering that demographic confusion may lead to bias, we limited the genetic background of the MR study population to individuals of European origin. The raw data for PD came from the publicly accessible GWAS dataset (GWAS ID: finn-b-F5_PANIC, https://gwas.mrcieu.ac.uk/datasets/finn-b-F5_PANIC/). In this dataset, 200,496 Europeans (2386 PD cases and 198,110 controls) were analyzed and 16,380,395 single nucleotide polymorphisms (SNPs) were acquired. The raw AD data came from IEU Open GWAS project (GWAS ID: ieu-b-5067, https://gwas.mrcieu.ac.uk/datasets/ieu-b-5067/ ) [27]. The data set included 488,285 Europeans (954 cases and 487,331 controls) and 12,321,875 SNPs.

### Selection of genetic instrument variables (IVs)

To screen for eligible gene IVs that meet the three key MR hypotheses, we have carried out a series of quality control procedures. First, independent SNPs closely associated with PD were selected where the *p*-value was less than 1*10^^−5^ [28]. Second, when the target SNPs are not in the final data set, they were replaced by proxy SNPs which have a strong linkage disequilibrium with the target SNPs. Third, *p* < 5*10^^−8^ was used to exclude the SNPs related to the outcome. Fourth, to exclude SNPs with strong linkage disequilibrium, we take R^2^ < 0.001, kb > 10,000 as the standard, which is derived from the European ancestral individuals of the 1000 Genomes Project [29]. Finally, the PhenoScanner was utilized to detect SNPs that may be linked to confounding factors with a threshold of *p* < 1*10^^−5^ [30].

### MR analysis

In this study, we used a variety of complementary approaches, including inverse variance weighting (IVW) and MR-Egger regression, weighted median and weighted mode methods, and MR-robust adjusted profile scores (MR-RAPS), for estimating the causal effects of PD on AD. The IVW method was mainly applied for basic causality estimation, which provided the most accurate results while all of the chosen SNPs were valid IVs. The IVW method calculated the weighted mean of the estimated Wald ratio [31]. With the assumption of instrument Strength Independent of Direct Effect (InSIDE), MR-Egger regression performed a weighted linear regression and yielded coherent causal estimates, even if genetic IVs were not valid. Yet, it showed poor accuracy and was susceptible to external genetic variation [32]. The weighted median regression method did not need the InSIDE assumption, and it calculated the weighted median of the Wald ratio estimation, which was robust to directional pleiotropy bias. The weighted median approach proved to have some strengths compared to MR-Egger regression, since it provided a lower type I error and higher efficacy of causality estimates [33]. The weighted mode approach estimates the causality of the subset with the greatest amounts of SNPs through grouping SNPs into subsets based upon causal similarity [34]. Finally, MR-RAPS used robust adjusted contour scores to correct directional pleiotropy, thereby reducing the deviation caused by directional pleiotropy [35].

### Sensitivity analysis

MR-Egger regression was performed to assess the likelihood of directional pleiotropy. The intercept of MR-Egger regression could indicate the average multiplicity of IVs [32]. In addition, the MR Pleiotropy REsidual Sumand Outlier (MR-PRESSO) test was carried out to assess the existence of directional pleiotropy. Its functions included testing for directional pleiotropy, rectifying directional pleiotropy by the removal of outliers, and ascertaining if the causal effects had changed substantially both before and after removing the abnormal values [36]. We used IVW method and MR-Egger regression to quantify the heterogeneity through the Q statistics of Cochran. The leave-one-out analysis was utilized to test the robustness and coherence of the conclusions. In addition, F-statistics were computed to assess for sample overlap effects and for weak instrumental deviations, with the formula below: F = R^2^*(N-2) / (1-R^2^). R^2^ represents the exposure variance interpreted by every IV. IV having an F-statistic lower than 10 was regarded as a weak instrument and was to be excluded from the MR analysis [37].

Estimates of causality were presented with odds ratios (OR) and 95% confidence intervals. All analyses were performed with “TwoSampleMR [38]” and “MRPRESSO” packages, R version 34.4.0.

## Results

The details of the SNPs identified in PD and AD are demonstrated in the Table 1. And from the Table 2, we could conclude that no evidence of heterogeneity of IV effect was found in Cochran’s Q test based on MR Egger and IVW, so the fixed effect model was used in MR analysis.

**Table 1:** Details of the SNPs identified in Panic disorder and Alzheimer’s disease

SNPs					Exposure (Panic disorder)				Outcome (Alzheimer’s disease)			
RS ID	Chr	Position	EA	OA	Beta	Se	pval	EAF	Beta	Se	pval	EAF
rs12439440	15	36,712,601	T	C	0.1369	0.0309	9.45E-06	0.3803	-6.31E-05	0.000103647	0.55	0.27687
rs12996926	2	83,298,520	C	T	0.2986	0.0659	5.80E-06	0.94275	0.000589608	0.000265881	0.0269998	0.967091
rs17138385	16	5,736,726	T	C	0.2	0.0425	2.52E-06	0.1502	0.000101696	0.000142594	0.48	0.129211
rs17505402	4	151,843,426	T	G	0.1566	0.0347	6.38E-06	0.2473	0.000150147	0.000119543	0.2	0.183658
rs17638240	7	41,764,955	C	T	0.3658	0.0782	2.90E-06	0.95987	0.000347692	0.000194666	0.0749998	0.939889
rs2975659	8	10,131,636	G	A	0.1856	0.0404	4.29E-06	0.1693	0.000244413	0.000139883	0.0779992	0.125874
rs2978487	8	23,416,034	T	C	0.1487	0.0319	3.09E-06	0.328	2.69E-05	9.69E-05	0.77	0.354657
rs35310982	17	41,388,787	G	T	0.513	0.1097	2.91E-06	0.02055	0.000281779	0.000304267	0.36	0.024864
rs7184371	16	61,940,564	G	A	0.1485	0.03	7.59E-07	0.4646	0.000103892	9.46E-05	0.27	0.399138
rs7254556	19	405,234	G	A	0.1442	0.0318	5.60E-06	0.6441	3.48E-05	9.92E-05	0.73	0.643028
rs76728418	9	10,241,374	C	T	0.2783	0.0598	3.23E-06	0.06788	0.00024731	0.00020415	0.23	0.054921
rs77676924	3	132,597,194	A	G	0.7001	0.1429	9.62E-07	0.01259	-3.12E-05	0.000234427	0.89	0.040562
rs804449	12	119,996,819	A	G	0.1814	0.0389	3.13E-06	0.1834	9.52E-05	0.000119426	0.43	0.18523

*SNPs* Single Nucleotide Polymorphisms, *Chr* Chromosome, *EA* Effect Allele, *OA* Other Allele, *Se* Standard Error, *pval* p_value, *EAF* Effect Allele Frequency

**Table 2:** Sensitivity analysis of MR analysis

Outcome	Heterogeneity tests				Directional horizontal pleiotropy				
	MR Egger		IVW		MR-Egger			MR-PRESSO global pleiotropy test	
	Q	pval	Q	pval	Intercept	Se	pval	RSSobs	pval
Alzheimer’s disease	9.866	0.542	10.004	0.616	2.85e-05	7.65e-05	0.717	12.830	0.611

*MR* Mendelian Randomization, *IVW* Inverse Variance Weighting, *Q* Cochran’s Q test, *pval* p_value, *Se* Standard Error

**Fig. 2:**
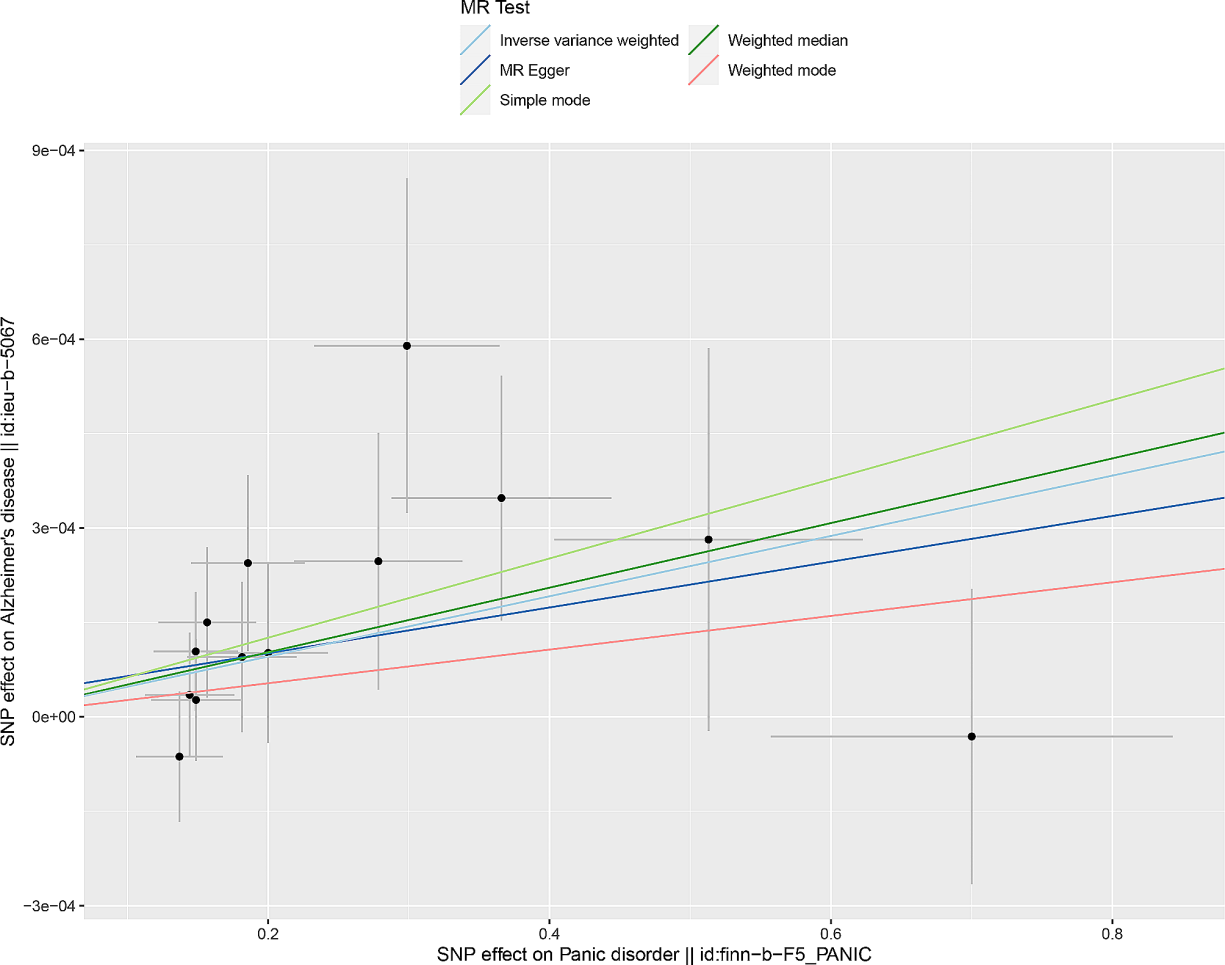
Scatter plot of causality. The slope of each line corresponds to the estimated MR effect in a different model

**Fig. 3:**
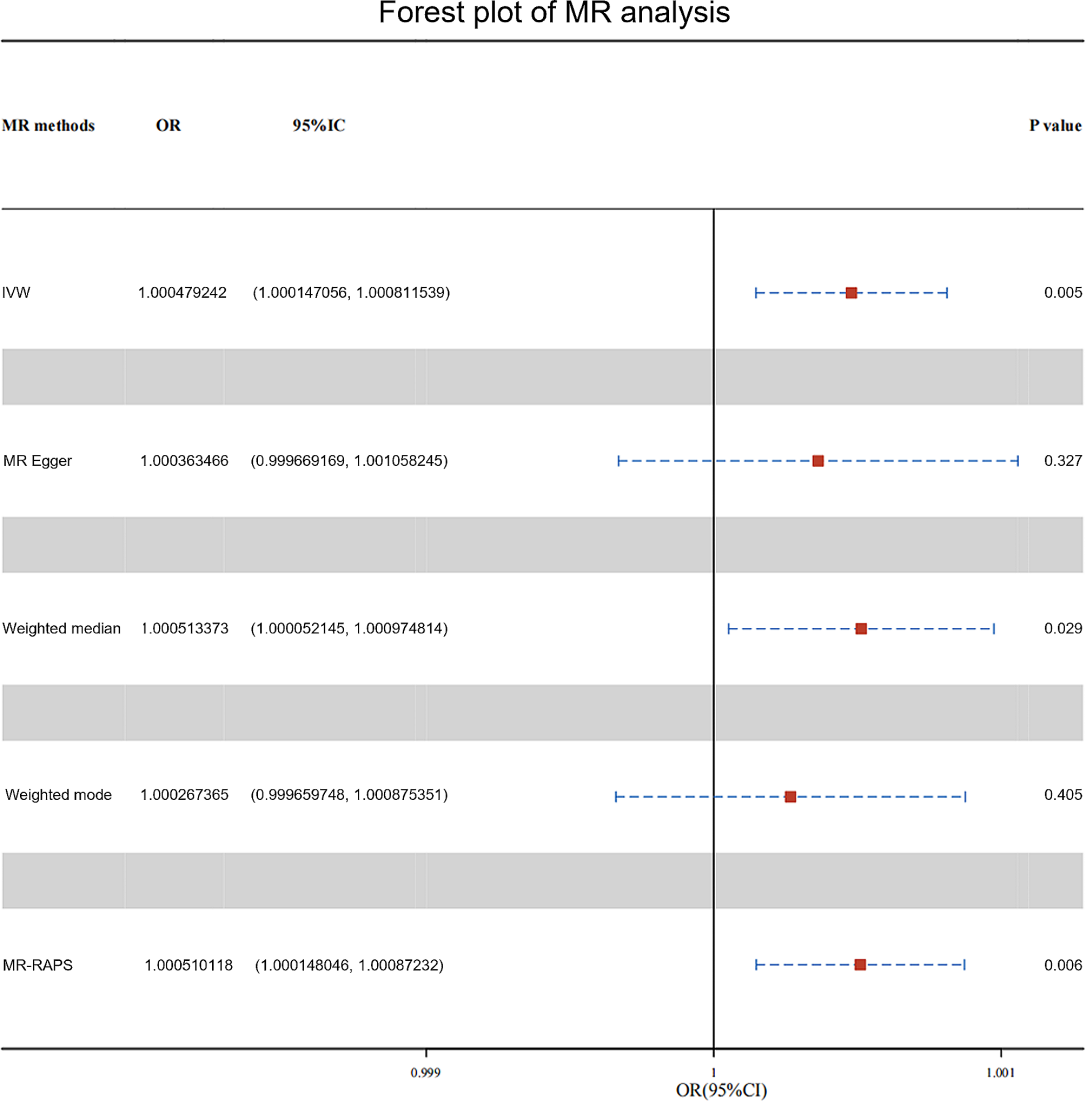
Detailed estimates of causal effects of the association between PD and AD in different models

**Fig. 4:**
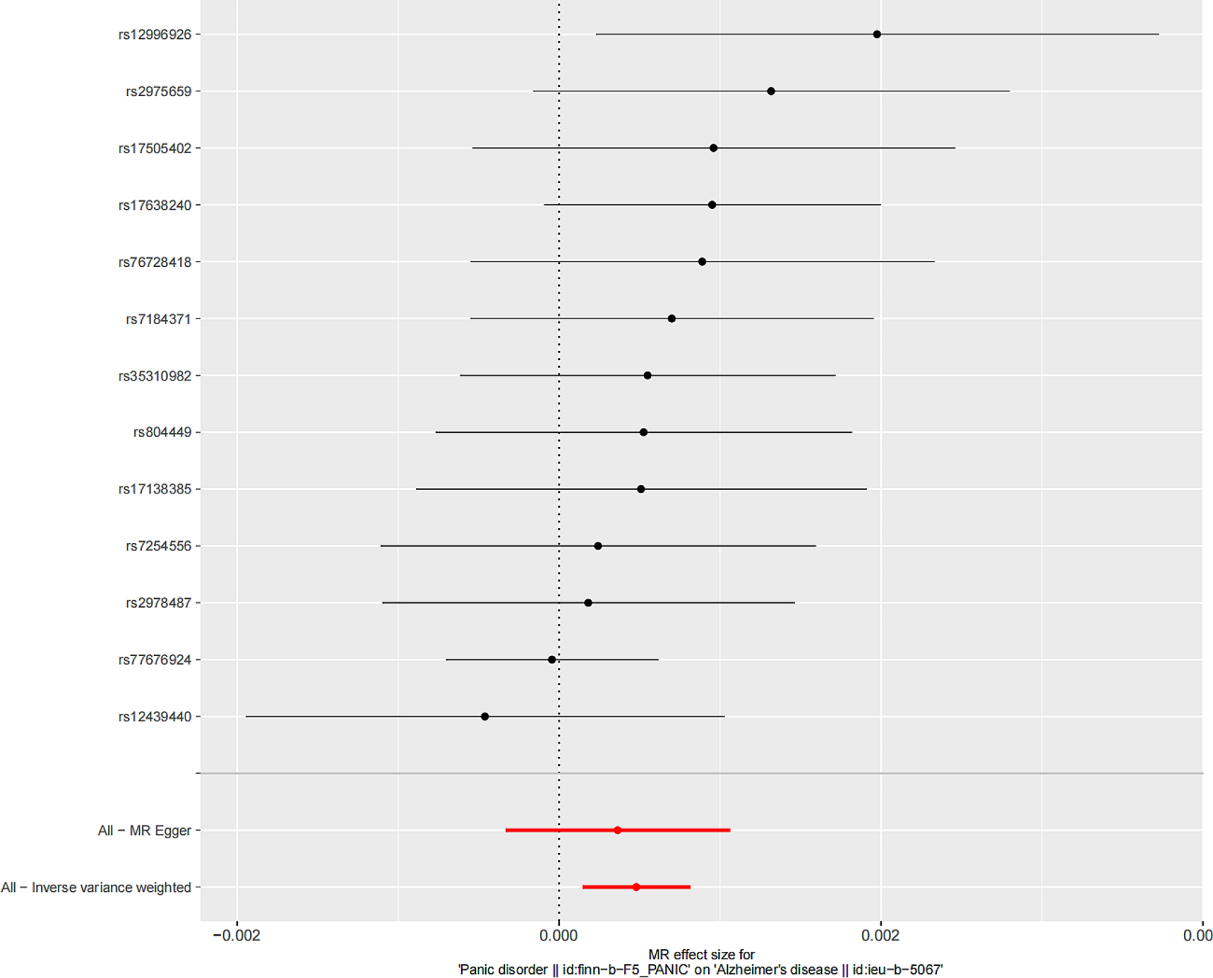
Detailed forest maps of MR effects for each IV in the IVW model

The causality estimated by the six models are shown in Figs. 2 and 3 and Supplementary Table 1. IVW analysis found that PD was related to an increasing risk of AD, and there was a causal correlation between them. And the detailed forest maps of MR Effects for each IV in the IVW model could be seen in the Fig. 4. At the same time, we verified this conclusion by weighted median and MR-RAPS.

**Fig. 5:**
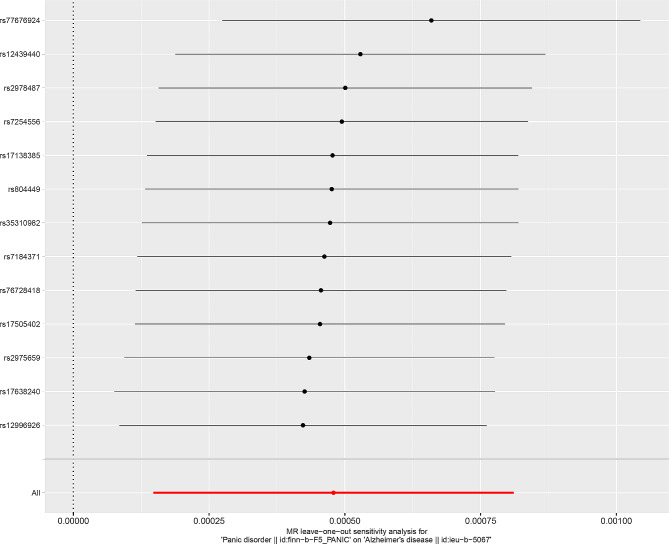
Detail diagram of the leave one out of sensitivity tests. The residual IVs were removed one by one and the MR Results of the remaining IVs were calculated

Then, we conducted extensive sensitivity analysis to verify the causality of PD and AD. The overall F statistic of the IVs was greater than 10. And R^2^ was lower than 0.001, indicating that there was no weakness in the selected tool. Both MR-Egger regression and MR-PRESSO global test showed no significant directional pleiotropy between PD and the risk of AD (Table 2). Finally, sensitivity tests showed the causality effect was not motivated by a sole IV, confirming the robustness of the results (Fig. 5).

## Discussion

We firstly utilized MR to systematically investigate the underlying causality of PD and the risk of AD. In the present study, we applied multiple complementary methods such as IVW to estimate the causal effect of PD on AD and 13 SNPs were identified. Then, the results of multiple MR analyses suggested that PD was related to an increasing risk of AD and that there was a potential causal relationship between them. Meanwhile, we also conducted sensitivity tests such as multiplicity analysis and heterogeneity analysis to exclude the interference of confounding factors and to ensure the accuracy and robustness of the MR results. In conclusion, the findings of this study implied that the genetic risk of PD was directly related to AD, and that early prevention and clinical intervention for AD should be provided to the PD population.

Previously, many observational studies or reviews indicated an association between PD and dementia. A recent report suggested that individuals with dementia may experience recurrent panic attacks as an initial symptom [39, 40]. In a prospective clinical study, it was observed that older adults with both depression and PD experienced accelerated memory decline compared to those without PD. Additionally, individuals with late-onset PD exhibited poorer cognitive abilities overall [41]. Numerous studies have consistently demonstrated that the presence of PD was associated with poor cognitive performance, including attention, memory, executive function, and spatial learning [18, 42–45]. These findings suggest that PD may contribute to the development of dementia or cognitive dysfunction.

Similar biological mechanisms may contribute to the causal link between AD and PD. Firstly, AD was closely related to dysfunction in both the prefrontal cortex and hippocampus, and lower volumes of both predict the development of AD [46]. Similarly, patients with PD exhibited hypoactivity in the prefrontal cortex and dysfunction in the hippocampus and amygdala [47–49]. Furthermore, the researchers found that reduced volume of the amygdala and hippocampus was a better predictor of dementia risk than hippocampal volume alone [50]. Secondly, the 5-HT1A receptor has been identified as being closely associated with the pathophysiology of PD and AD and this receptor is considered a common target for drug treatment in both diseases [19–21]. Thirdly, PD and AD have been found to share multiple differentially expressed genes, including Adrenoceptor Alpha (ADRA2A, ADRA2C), Glutamate Ionotropic Receptor (GRIN3A, GRIN2B, GRIN2C, GRIN2D), and Monoamine Oxidase A (MAOA) [51].

Anxiety has been implicated in potentially causing neurological damage and having a negative impact on cognition, as indicated by previous studies. Several hypotheses have been proposed to explain this connection: (1) Hypercortisolemia caused by anxiety may lead to hippocampal atrophy [52–55]; (2) Anxiety can cause cardiovascular disease, low-grade inflammation, or decreased levels of brain-derived neurotrophic factor [56–58]. These may explain the causal association between anxiety and AD or cognitive function. While PD is a common subtype of anxiety, the specific causal link between PD, AD, and cognitive impairment requires further exploration.

Nevertheless, some of the studies had yielded contradictory results. A cross-sectional study (*N* = 7344) found that current PD was significantly associated with memory but not with executive function processing speed or cognitive impairment [18]. Other studies showed that anxiety disorders, including PD, were not found to increase the risk of dementia [17, 59]. Briefly, observational studies alone make it difficult to investigate the causality of PD and AD. Therefore, in the present study, we innovatively used MR analysis to demonstrate a statistically significant causal effect between PD and AD, while also eliminating the interference of confounding factors. This finding was of great significance for the clinical management of these two diseases.

### Advantages

This study has several advantages. Firstly, this study was the first MR analysis study exploring the causality between PD and AD, which provided further insight into the genetic knowledge of PD and AD. Secondly, it was worth pointing out that we used several different and complementary models to replicate the identification of causal effects between PD and AD risk, and reached the same conclusions. Thirdly, different from traditional observational studies, in this study we applied multiple methods, such as multivalent analysis, heterogeneity analysis and estimation of the effect of sample overlap on instrumental variables, to avoid the bias resulting from confounding factors and to guarantee the robustness and accuracy of the MR results.

### Limitations

The study also has some limitations. Firstly, to avoid potential confusion from a more heterogeneous population, the population we included in the MR analysis came from European origin,but this may limit the confidence for our results extended to other races. Secondly, overlapping participants may be present in the exposure and outcome studies, yet the extent of sample overlap could hardly be evaluated. Fortunately, the use of powerful tools in this study (for example, F statistics greater than 10) had minimized the potential deviation of sample overlap [60]. Finally, it is worth pointing out that in this study, although the causal hazard ratio between PD and AD was found to be statistically significant, the ratio was very close to 1, which may indicate that there might be more of a biological correlation between the two, rather than a firm causal relationship. The results therefore need to be interpreted with caution, especially in clinical practice and decision-making. In addition, in order to better understand the interaction between PD and AD, first, it is suggested to further investigate the causal relationship between PD and AD in different ethnic groups. Second, in addition to genetics, it is proposed to further explore the underlying biological mechanisms of causality between PD and AD.

## Conclusion

In conclusion, our findings demonstrate that PD may increase the risk of AD from a genetic point of view. This contributes to our understanding of the genetic association between the two disorders, and also provides clinicians with new ideas for the diagnosis and treatment of patients with PD and AD. When treating patients with PD or patients with a history of PD, clinicians should focus on their AD symptoms, which may help in choosing treatment measures or reducing comorbidities. In addition, exploring medications that both treat PD and improve AD would better advance clinical care; however, further clinical research is needed to replicate these findings and investigate the efficacy of treating the comorbidity of PD and AD. Further research is needed to elucidate the underlying mechanisms of comorbidity.

### Electronic supplementary material

Below is the link to the electronic supplementary material.

Supplementary Material 1

## Data Availability

All the datasets analyzed during the current study are publicly available in the GWAS summary data repository. Raw datasets for panic disorder can be downloaded at FinnGen base (https://gwas.mrcieu.ac.uk/datasets/finn-b-F5_PANIC/ ). Raw datasets for Alzheimer disease can be downloaded at IEU Open GWAS project (https://gwas.mrcieu.ac.uk/datasets/ieu-b-5067/ ) [27]. More details are available from the corresponding author on reasonable request.
